# Natural Killer p46 Controls Hepatitis B Virus Replication and Modulates Liver Inflammation

**DOI:** 10.1371/journal.pone.0135874

**Published:** 2015-08-20

**Authors:** Wanyu Li, Yanfang Jiang, Xiaomei Wang, Jinglan Jin, Yue Qi, Xiumei Chi, Hong Zhang, Xiangwei Feng, Junqi Niu

**Affiliations:** Department of Hepatology, First Hospital, Jilin University, No. 71 Xinmin Street, Changchun, 130021, China; CRCL-INSERM, FRANCE

## Abstract

Natural killer (NK) cells play an important role in hepatitis B virus (HBV) infection control, and are regulated by a complex network of activating and inhibitory receptors. However, NK cell activity in HBV patients remains poorly understood. The objective of this study was to investigate the phenotypic and functional characteristics of circulating NK cells in patients during different chronic hepatitis B (CHB) infection stages. We investigated NK cell phenotypes, receptor expression and function in 86 CHB patients and 20 healthy controls. NK cells were purified and NK cell subsets were characterized by flow cytometry. Cytotoxic activity (CD107a) and interferon-gamma (IFN-γ) secretion were examined, and Natural Killer p46 (NKP46) blockade and spontaneous NK cell cytolytic activity against K562, HepG2 and HepG2.215 cell lines was studied. Activating NKp46 receptor expression was higher in inactive HBsAg carriers when compared with other groups (*p =* 0.008). NKp46 expression negatively correlated with HBV DNA (R = -0.253, *p* = 0.049) and ALT (R = -0.256, *p* = 0.045) levels. CD107a was higher in immune-activated groups when compared with immune-tolerant groups (*p =* 0.039). CD107a expression was related to viral load (*p* = 0.02) and HBeAg status (*p =* 0.024). *In vitro* NKp46 blockade reduced NK cell cytolytic activity against HepG2 and HepG2.215 cell lines (*p* = 0.02; *p* = 0.039). Furthermore, NK cells from high viral load CHB patients displayed significantly lower specific cytolytic activity against anti-NKp46-loaded K562 targets (*p =* 0.0321). No significant differences were observed in IFN-γ secretion (*p* > 0.05). In conclusion, NKp46 expression regulates NK cell cytolytic function. NKp46 may moderate NK cell activity during HBV replication suppression and HBV-associated liver damage and may be critical for NK cell activity during CHB infection.

## Introduction

Hepatitis B virus (HBV) infection is a major global health concern that affects approximately 240 million people worldwide [1,2]. HBV infections follow a course that is divided into four phases: immune tolerance, immune clearance, low replication and reactivation [3]. Because HBV is not cytopathogenic, the host immune responses induced by viral persistence are generally thought to be responsible for disease progression in chronic HBV (CHB) patients [4]. Additionally, because acute and CHB infections involve different immune responses, a complete understanding of the diversity of CHB infections and host immune responses remains elusive [5]. It is generally accepted that HBV-specific T cells play an important role in CHB infection induced hepatocellular damage [6,7]; however, recent studies report that other innate immune effector mechanisms may be responsible for viral clearance and liver pathogenesis [3].

Natural killer (NK) cells are an important component of the innate immune system, and NK cell-deficient individuals may suffer from repeated viral and bacterial infections. It is known that NK cells kill virus-infected cells; however, insufficient knowledge concerning NK cell immunobiology has impeded research seeking to understand the role of these cells in infection control [7]. Unlike the well-defined single antigen receptors of T and B cells, NK cells express a diverse and distinct class of activating or inhibitory NK receptors (NKR) that are capable of binding a variety of ligands. NK cell expression of the NKp46 surface receptor is conserved across mammalian species, and this has allowed the use of NKp46 as a reliable NK cell marker in various animals [8].

NK cells are abundant in the liver, where they serve as a major component of the liver’s innate immune system [4]. Recent studies indicate that NK cells are characterized by a functional dichotomy in chronic viral hepatitis patients. In these patients, NK cells both conserve and enhance cytotoxicity, and reduce interferon (IFN)-γ production [9,10]. This functional dichotomy is especially clear during HCV infection, where the NK cell subtype NKp46 appears to be involved in HCV replication suppression, antifibrotic activity and HCV-associated liver damage [11]. Supporting the importance of NKp46 and NK cells in the immunopathogenesis of HCV, a pair of elegant studies performed by the laboratories of Jacob Nattermann and Hugo Rosen found that NKp46^High^ expression defined a specific NK cell subset that might be involved in both HCV replication suppression and HCV-associated liver damage [11–13]. However, for HBV this remains a subject of debate. In animal models of HBV infection, early intracellular immune responses are poorly induced by HBV because HBV behaves like a stealth virus and does not induce the innate immune system [14]. Other studies have challenged this view, arguing that HBV can be sensed by the innate immune system [15,16]. However, if the virus persists, activated NK cells can mediate hepatocyte apoptosis [3]. It has been reported that NK cells contribute to liver inflammation in CHB during inflammatory flares, and that NK cell activity in CHB can be affected by viral load [17]. Because HBV and HCV share similar natural histories, pathogenesis and transmission modalities, it is possible that NK cells, and NKp46 in particular, play a functional role during CHB infection [18].

Because NKRs play important roles in NK cell regulation, several studies have analyzed NK cell expression of inhibitory and activating NKRs during acute and CHB infections. Most of these studies have reported increased expression of the inhibitory receptor NKG2A and a number of activating receptors, including NKP30, NKG2D and NKp46 [3,9,19–22]. However, the phenotypes and functions of NK cells in HBV and CHB infections remain controversial and a significant amount of work remains to be done to understand the roles of NKRs and NK cells in CHB infections.

We previously reported a dramatic decrease in NK cell number in cirrhotic HBV patients, indicating a cirrhosis associated decrease in innate immunity [18]. In the present study, we investigated the phenotypic and functional characteristics of circulating NK cells in patients with CHB infections at different stages. While previous research has focused solely on the immune tolerance and immune reactivation phase, we observed, in this study, variations in NKp46 expression in the peripheral blood NK cells of patients during all four stages of CHB. Furthermore, we report that NKp46 expression is correlated with cytolytic activity, viral replication and liver inflammation.

## Methods

### Patients

Eighty-six Chinese patients and 20 controls from the First Hospital of Jilin University were enrolled in this study between September 2013 and September 2014. The patients enrolled in this study included representatives from each of the four HBV phases described in the introduction: immune-tolerant phase (24 chronic HBV carriers), immune-activated phase (24 HBeAg-positive CHB, 16 HBeAg-negative CHB) and low replication phase (22 inactive HBsAg carriers) (S1 Table). All patients were diagnosed according to previously described criteria [2,4,5].

Twenty age- and sex-matched healthy donors with no history of HBV or HCV infection were included as the control group. Exclusion criteria included: patients with human immunodeficiency virus, other types of hepatitis, autoimmune liver diseases, alcoholic liver disease, carcinoma or who received interferon and nucleoside or nucleotide analog treatments up to one year prior to enrollment. All protocols followed in this study conformed to the ethical guidelines of the 1975 Declaration of Helsinki, and were approved by the Institutional Review Committee of the First Hospital of Jilin University. Written informed consent was obtained from each participant enrolled in this study. Patient demographic characteristics and clinical features are summarized in Table 1. Peripheral blood mononuclear cells (PBMCs) were isolated from all enrolled subjects.

**Table 1:** Demographic Characteristics and Clinical Features.

	Healthy	Chronic	Inactive	HBeAg+	HBeAg-
	controls	HBV carriers	HBsAg carriers	CHB	CHB
number	20	24	22	24	16
Gender (male:female)	13:7	18:6	19:3	19:5	8:8
age‡	42.1 ± 10.9	32.8 ± 8.9	32.2 ± 7.4	34.8 ± 11.0	43.1 ± 11.0
ALT†	< 50	< 50	< 50	228.3 (56.0–479.9)	154.6 (52.3–504.0)
AST†	< 40	< 40	< 40	130 (42.1–445.0)	95.7 (51.0–232.0)
HBV DNA‡ (log10copies/ml)	< 3	7.0 ± 1.8	< 3	6.9 ± 1.7	5.2 ± 1.7

Normal values: alanine aminotransferase (ALT), ≤ 40 IU/L; aspartate aminotransferase (AST), ≤ 40 IU/L; HBV DNA ≤ 3 log_10_ copies/ml

^†^Data are expressed as median (range)

^‡^Data are expressed as means ± SD.

### Flow cytometry

Phycoerythrin (PE)-conjugated anti-NKG2C, anti-KIR2DL3 and anti-KIR3DL1 antibodies were purchased from R&D Systems (Minneapolis, MN, USA). All other antibodies were purchased from BD Biosciences (San Jose, CA, USA). Peripheral NK cell subset frequencies and NKR expression levels were analyzed according to previously described protocols [2,3]. Samples were analyzed using a FACSCanto flow cytometer and the CellQuest Pro (BD Biosciences) and FlowJo 7.2.2 software packages (TreeStar Inc., Ashland, OR, USA).

### IFN-γ secretion

PBMC cells were cultured at 37°C and 5% CO2 in the presence or absence of recombinant human interleukin (IL)-12 (0.1–10 ng/mL; eBioscience, San Diego, CA, USA) for 16 hrs. Next, brefeldin A (BFA; 10 g/mL; Sigma-Aldrich, St. Louis, MO, USA) was added and the cells were incubated for another 4 hrs. Finally, intracellular staining was performed using anti- IFN-γ and FACS analysis was conducted according to previously described protocols [2,18,19].

### CD107a degranulation detection

CD107a degranulation is widely used to assess the cytotoxic potential of NK cells. Briefly, freshly isolated PBMCs (1 x 10^6^) were stimulated with propylene glycol monomethyl ether acetate (PMA; 100 ng/mL-1) and ionomycin (1 mg/mL-1), IL-12 (10 ng/mL-1) or K562 cells at an effector to target ratio (E:T) of 10:1. Unstimulated PBMCs served as negative controls. Simultaneously with the above treatments, Anti-CD107a was added to the medium and incubated for 5 h. Cells were collected and immediately stained with surface antibodies (no fixation was conducted prior to staining) [23].

### NK cell separation

MACS cell separation kits were used according to the manufacturer’s recommendations to immunomagnetically separate NK cells from total PBMCs *via* non-NK cell depletion (BD Biosciences). NK cell purity was >95%.

### NKp46 in vitro blockade

NK cells and PBMCs were pre-incubated at 37°C for 24hrs with anti-NKp46 (10 ug/mL; R&D Systems) or isotype control (10 ug/mL; R&D Systems). Next, PBMCs from high viral load CHB patients and healthy controls were incubated with K562 cells at an E:T of 10:1. Human hepatoblastoma derived HepG2 and HepG2.215 cells were cultured as previously described [24]. Healthy control NK cells were incubated with the hepatocellular carcinoma cell lines (HepG2 and HepG2.2.15 respectively) at E:Ts of 1:1, 5:1, 10:1 and 20:1. NK cell killing activity was analyzed using a lactate dehydrogenase (LDH) cytotoxicity assay kit (Promega, Madison, WI, USA) according to manufacturer’s instructions [25].

### Virological assessment

The virological assay was performed as previously described with a cut-off value of 1000 copy/ml [18].

### Statistical analysis

All clinical and flow cytometry data were compared using Wilcoxon rank sum and Chi-square tests. Correlations were determined using Spearman's correlation test. SAS version 9.0 software (SAS Institute Inc., Cary, NC, USA) was used for all analysis. Results are reported as median (range), unless otherwise specified. A two-sided *p* value < 0.05 was considered significant.

## Results

### NKp46 was enriched in inactive HBsAg carriers

Analysis of the proportions of circulating NK cells (CD3^-^CD56^+^) and NK cell subtypes (CD3^-^CD56^bright^ and CD3^-^CD56^dim^) in all enrolled subjects revealed no significant differences between these groups (*p* > 0.05).

**Fig 1:**
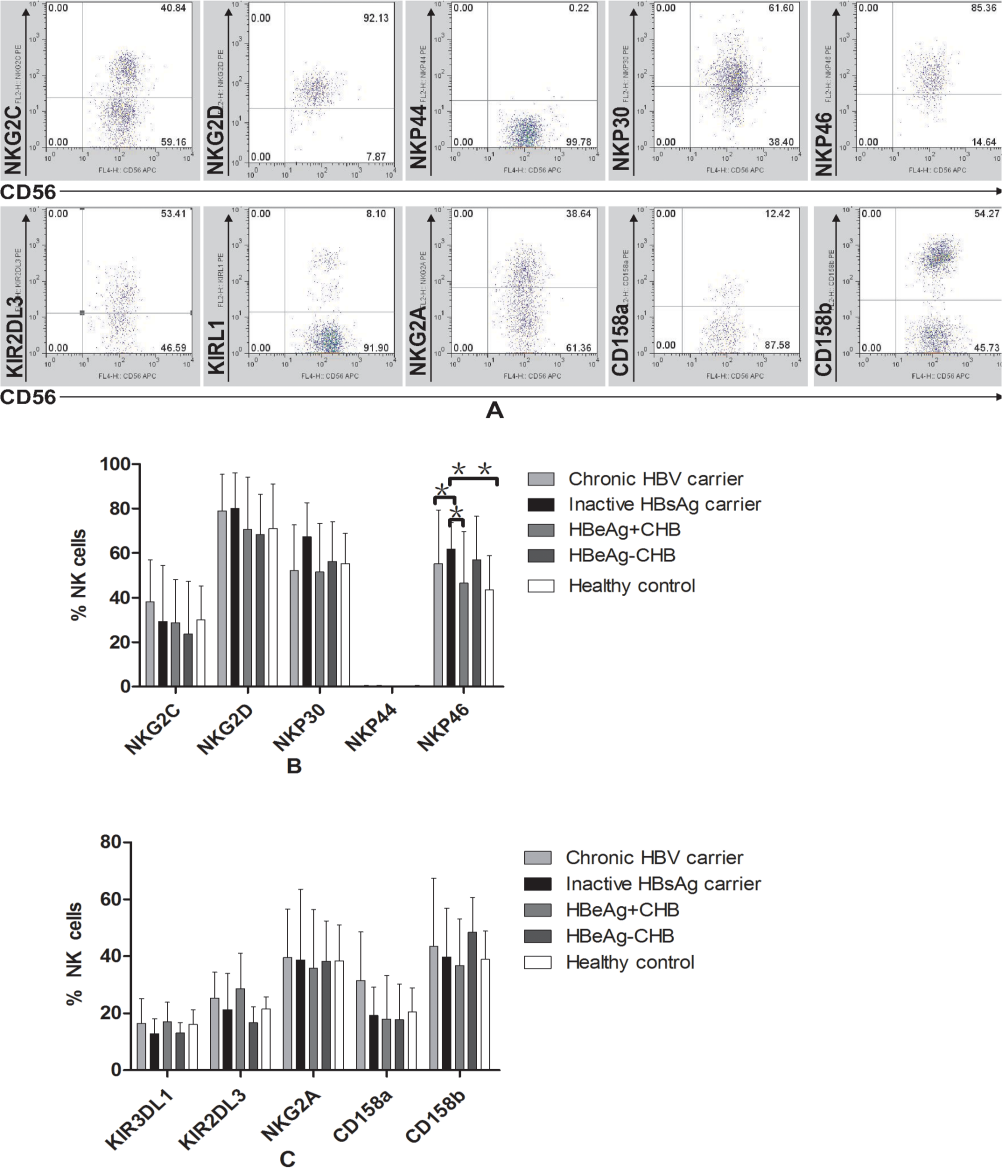
NK cells display abnormal NK receptor expression in CHB patients. (A) Representative dot plots depict NK activation receptor expression (NKp30, NKp44, NKp46, NKG2D and NKG2C) and inhibitory receptors (CD158a, CD158b, KIR2DL3, KIRL1 and NKG2A) in a CHB patient. Pooled data show the proportion of (B) NK cells expressing the NK activation receptors NKp30, NKp44, NKp46, NKG2D and NKG2C and (C) NK cells expressing the NK inhibitory receptors CD158a, CD158b, KIR2DL3, KIRL1 and NKG2A in HC subjects (n = 20), HBeAg-positive CHB subjects (n = 24), HBeAg-negative CHB subjects (n = 16), chronic HBV carriers (n = 24) and inactive HBsAg carriers (n = 22). The data represent the means ± SD. *p < 0.05, **p < 0.01.

We analyzed CD3^-^CD56^+^ NK cell NKR expression in the four CHB phase groups (including activating receptors NKG2C, NKG2D, NPK44, NKP30 and NKp46, and inhibitory receptors NKG2A, CD158a, CD158b, KIR2DL3 and KIR3DL1; Fig 1A, S2 Table). We found that NKp46 was elevated in inactive HBsAg carriers when compared with patients from other groups (Fig 1B, S2 Table). No significant expression differences were observed for any of the other receptors between any of the CHB phase groups (Fig 1C, S2 Table).We also examined NK cell IFN-γ production, but no differences were observed in any of the CHB patient groups (*p* > 0.05).

### NKp46 expression negatively correlated with HBV DNA and alanine aminotransferase (ALT)

**Fig 2:**
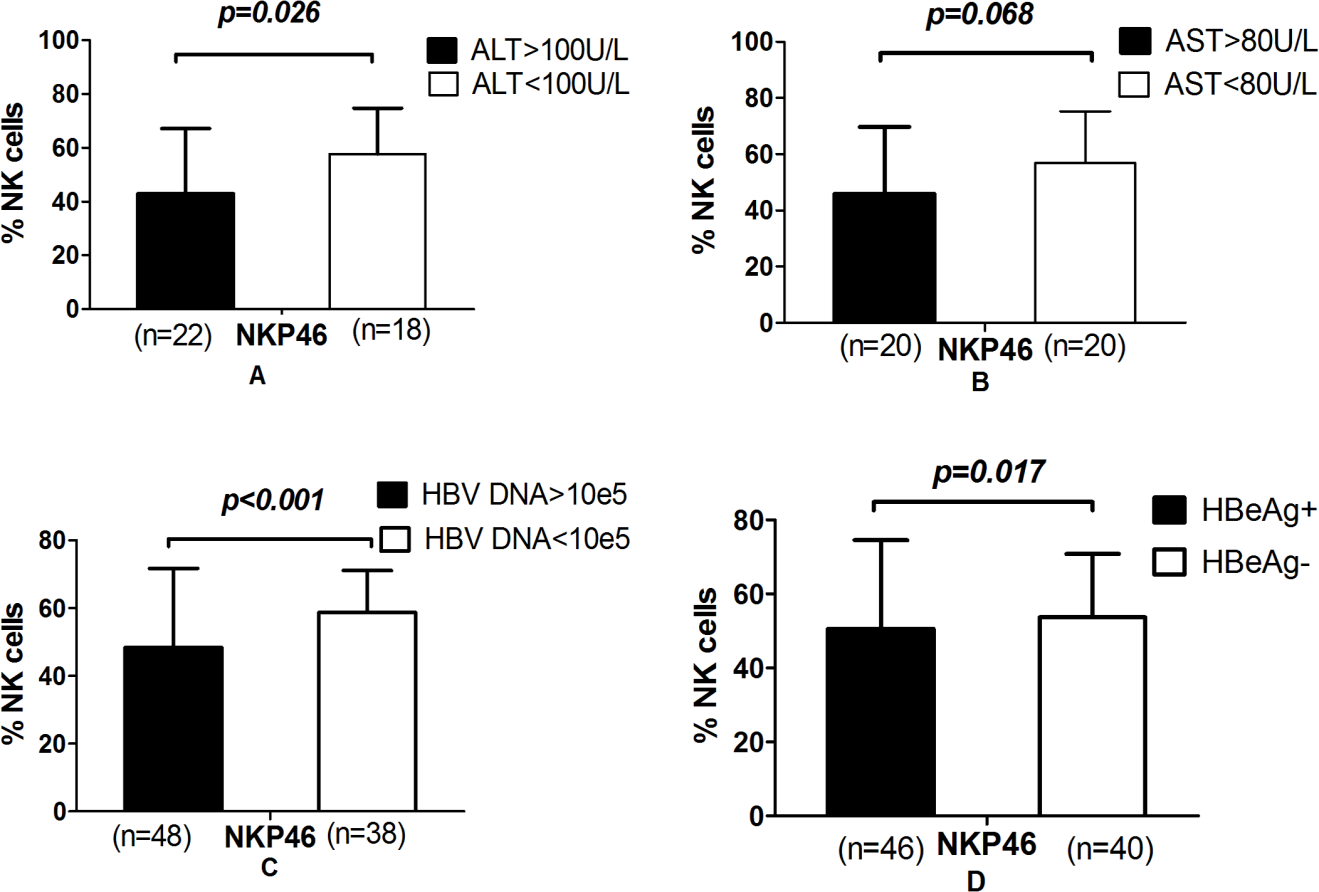
NKp46 was decreased in the high alanine aminotransferase, high viral load and HBeAg positive groups. (A) Groups divided by ALT level. (B) Groups divided by AST level. (C) Groups divided by HBV DNA levels. (D) Groups divided by HBeAg status.

Analysis of the correlations between NK cell receptor expression and the clinical parameters (such as viral load and aminotransferase levels) of CHB patients indicated that NKp46 expression negatively correlated with HBV DNA (R = -0.253, *p* = 0.049) and ALT (R = -0.256, *p* = 0.045) levels. For this analysis, patients were divided into groups according to the following clinical phenotypes: HBeAg-positive or HBeAg-negative, high viral load or low viral load (> 5 log_10_ copies/ml or < 5 log_10_ copies/ml, respectively), high or low ALT (> 100 units/L or < 100 units/L, respectively) and high or low aspartate aminotransferase (> 80 units/L or < 80 units/L, respectively). We observed a significant decrease in NKp46 expression in the high ALT, high viral load and HBeAg positive groups (Fig 2, S3 Table). None of the other NKRs examined exhibited significant relationships with HBeAg status, viral load or aminotransferase levels (*p* > 0.05).

### Circulating NK Cells have a strong cytotoxic potential in the immune-activated group

**Fig 3:**
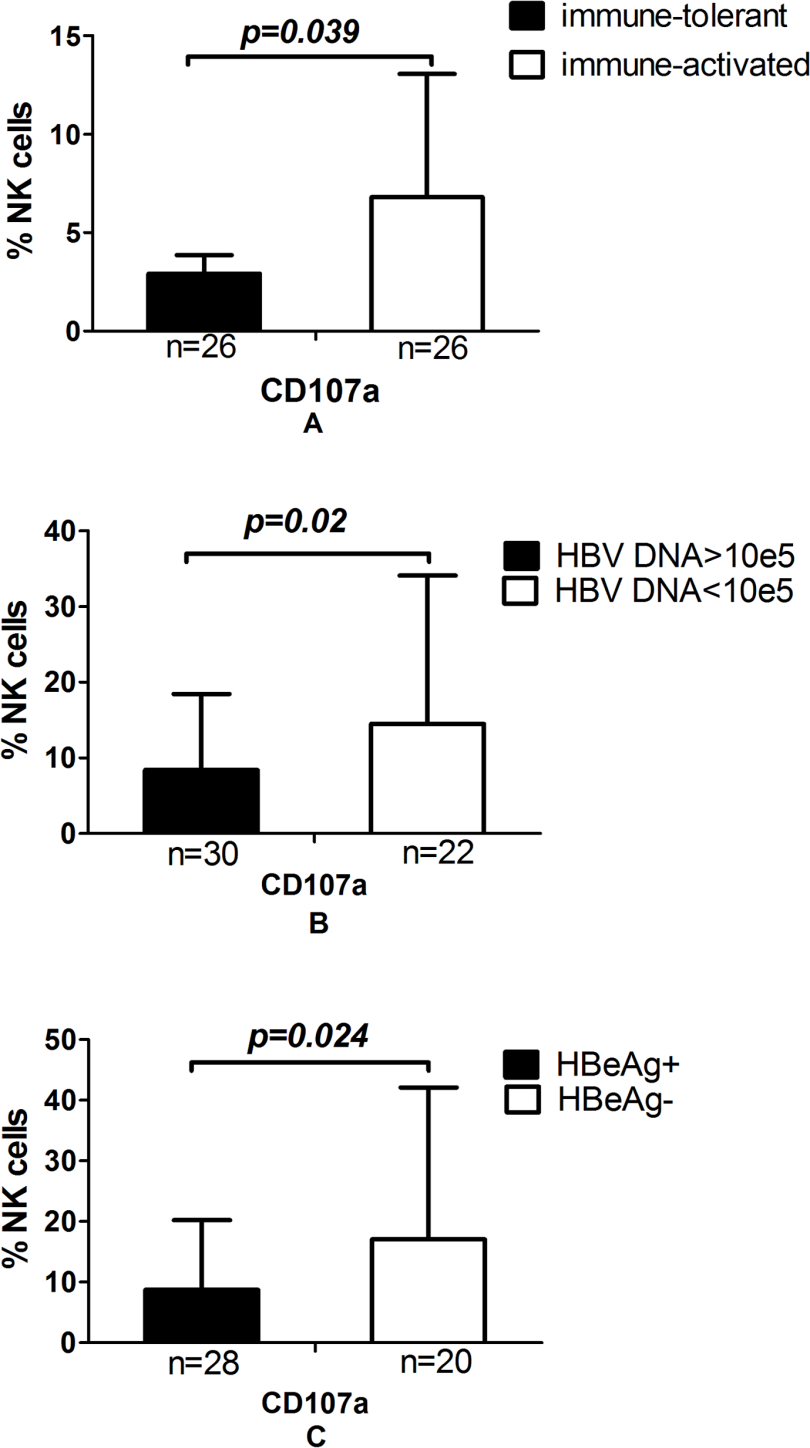
NK cell expression of CD107a was abnormal in CHB patients. (A) Groups divided by immune status. (B) Groups divided by HBeAg levels. (C) Groups divided by HBV DNA levels.

NK cells in the immune activated phase group displayed significantly higher cytolytic activity against K562 cells when compared with the immune tolerance phase group (Fig 3A, S4 Table). Importantly, cells with higher CD107a expression exhibited strong cytotoxic activity in the low viral load and HBeAg negative groups (Fig 3B and 3C, S4 Table).

### NKp46 is involved in the cytotoxic activity of NK cells

**Fig 4:**
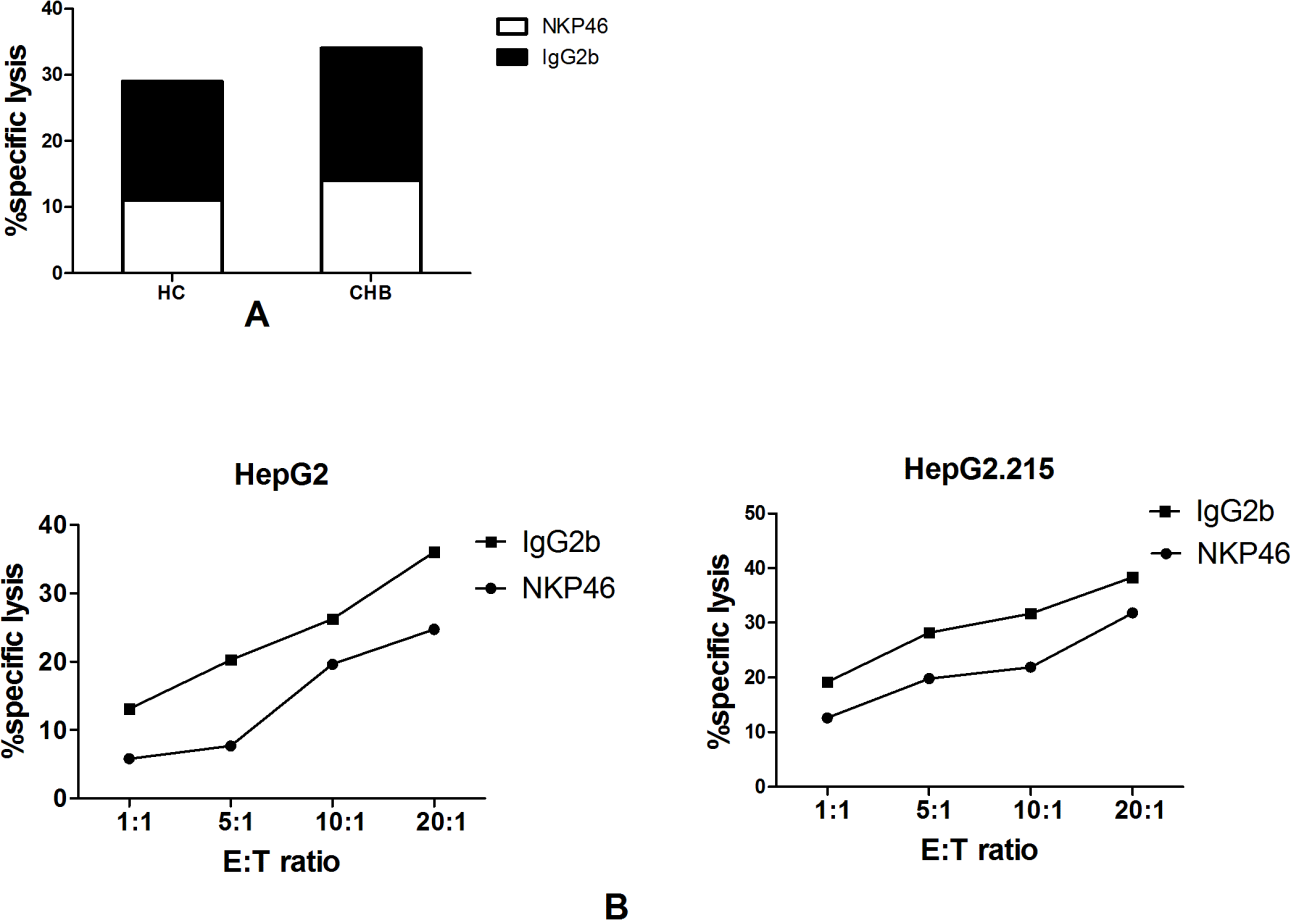
NKp46 is involved in the cytotoxic activity of NK cells. (A) NK cells displayed significantly lower specific cytolytic activity against hepatoma cell lines and (B) K562 *in vitro* blockade. Effector to target ratio (E:T).

We analyzed the NKp46-specific cytotoxic activity of NK cells after blocking NKp46 activity *in vitro*. NK cells from high viral load CHB patients displayed significantly lower specific cytolytic activities when compared with anti-NKp46-loaded K562 cells (*p* = 0.0321; Fig 4A, S5 Table). However, no significant difference was observed between the healthy control group and anti-NKp46-loaded K562 cells (Fig 4A, S5 Table).

Additionally, we observed the NKp46-specific cytotoxic activity of NK cells using anti- NKp46 and the HepG2 and HepG2.215 target cell lines. Anti- NKp46 blockaded NK cells displayed a significantly lower specific cytolytic activity against the hepatoma cell lines (HepG2, *p* = 0.02; HepG2.215, *p* = 0.039; Fig 4B, S5 Table)

## Discussion

Evidence is accumulating that NK cells play a critical role in HBV infection control. For instance, immunogenetic analyses indicate that NK cells influence HBV infection outcomes [4]. Recent studies have indicated that NK cells may influence the immunopathogenesis of CHB by demonstrating that activated NK cells preferentially accumulate in the livers of immune-activated patients and that elevated NK cytolytic activity is associated with liver injury [3]. However, because current studies have yielded inconsistent results regarding the phenotypes and functions of NK cells, complete understanding of the role of NK cells in HBV infected patients remains elusive [26].

The inconsistent results regarding NK cell phenotypes may have been caused by methodological issues or by differences in the characteristics of the study cohorts. We hypothesize that a distinct natural immunological milieu exists in each CHB phase. Therefore, it is critical that future investigations include comprehensive data regarding the immune stages and disease spectrums of the CHB patients enrolled in the study. Additionally, because NKR expression levels differ during the distinct immune stages of CHB, further research is needed concerning the differential expression of NKRs. In this study, we investigated the roles played by different NK cell subpopulations and NKRs in CHB.

We first compared the *ex vivo* functional and phenotypic characteristics of circulating NK cells. We examined the major activating receptor NKp46 and found that its expression was higher in the low replication phase when compared with the immune-tolerance phase, immune-clearance phase and healthy control groups. Our results are consistent with a previous study [21], and support the hypothesis that, when compared with low replication phase, immune-tolerance patients are characterized by a high viral load, normal ALT levels and a low endogenous HBV immune response. In contrast, immune-clearance phase patients are characterized by a high viral load, abnormal ALT levels and a partially activated HBV immune response. Werners *et al*. observed elevated NKp46 levels in HCV exposed healthcare workers who did not develop acute infections [27]. Lorenzo *et al*. observed that Peg-IFNα induced significant increases in the percentage and absolute numbers of CD56^bright^ NK cells expressing NKP46 [28]. Therefore, elevated NKp46 may be an indicator of HBV or HCV remission.

In the current study, we also demonstrated an association between NKp46 expression, HBV viral load and ALT. NKp46 expression was significantly decreased in high ALT, high viral load and HBeAg positive patients. Our results indicate that NKp46 expression levels may be inversely correlated with hepatic inflammation and viral load. This is supported by a previous report in which therapeutic IFN-α was used to induce IL-15 and NKp46 expression. In that report, NKp46 expression induction was accompanied by the expansion and activation of NK cells capable of exerting antiviral effector functions [21]. Although it was a variant in the cytolytic activity of different CHB groups, our functional studies observed no differences in NK cell IFN-γ production. Thus, low viral load and hepatic inflammation may be conditions that favor NKp46 activation, and increased NKp46 expression after NK cell activation may support the inhibition of HBV replication and hepatic inflammation. This hypothesis is supported by the results of numerous investigations studying chronic HCV infection [11–13].

We analyzed the cytolytic activity of the NK cells of the different CHB groups *via* CD107a degranulation. In agreement with the results of Fusheng Wang *et al*., we found that CD107a expression was higher in the immune activated phase than the immune tolerance phase. This result indicates that NK cells are activated during the immune activated phase to facilitate viral clearance, and supports the hypothesis that NK cell cytotoxicity mediates hepatic injury [5]. Additionally, we observed significantly decreased CD107a levels in both the high viral load and HBeAg positive groups, which was in line with the results reported by Hairong Wei *et al* [17]. Therefore, we can infer that high viral replication may inhibit NK cell cytotoxicity.

In accordance with this inference, we examined high viral load CHB patients and observed both that NK cell cytotoxicity was decreased and that the number of NKp46+ NK cells was increased. This suggested that decreased NKp46 expression leads to reduced NK cell cytotoxicity. Therefore, we investigated the effect of an anti-NKp46 *in-vitro* blockade. Blocking NKp46 *in vitro* significantly reduced the cytotoxic capability of circulating NK cells against K562 and hepatoma cell lines, suggesting that NKp46 may be a major lytic receptor and directly involved in the regulation of NK cell cytotoxicity. Supporting our observations, a previous HIV infection study reported that NKp46 expression directly correlated with the degree of NK cell cytotoxicity [29].

One factor limiting this study was the small number of patient samples examined. Another limitation was the absence of *in vivo* experiments investigating NK cell function. Therefore, the *in vivo* effects of NKp46 on HBV replication and liver inflammation remain unknown. Although a great deal of research has been conducted exploring the role of NKRs in HBV infections, many of the results remain controversial. Therefore, the mechanisms underlying the differential regulation of NK activity in CHB patients are not yet fully understood and must be further investigated by future studies.

In summary, we present preliminary data indicating that NKp46 represents a major specific NK cell receptor. Furthermore, our findings indicate that NKp46 may be involved in viral replication control and the inhibition of liver inflammation in CHB. Moreover, our data clearly supports an important cytolytic role for the NKp46 receptor in HBV infections.

## Supporting Information

S1 Table Raw Data. (XLS)

S2 Table The expressions of NK receptors in CHB patients. (DOC)

S3 Table NKP46 receptor expression in different groups divided by ALT, AST, HBV DNA levels and HBeAg status. (DOC)

S4 Table CD107a expression in different groups divided by immune status, HBeAg levels and HBV DNA levels. (DOC)

S5 Table In vitro blockade of NKP46. (XLS)
