# Supplementary material for: Natural Killer p46 Controls Hepatitis B Virus Replication and Modulates Liver Inflammation
Source: PLoS One. 2015 Aug 20;10(8):e0135874. doi: 10.1371/journal.pone.0135874 (PMC4546267; doi:10.1371/journal.pone.0135874)
Supplement: S2 Table — (DOC) [file pone.0135874.s002.doc]

Table S2.Data show the proportion of NK cells expressing the NK activation receptors and inhibitory receptors,IFN-gamma and CD107a.

|  | Healthy  controls | Chronic  HBV  carriers | Inactive  HBsAg  carriers | HBeAg+  CHB | HBeAg-  CHB |
| --- | --- | --- | --- | --- | --- |
| NKG2C  (% NK cells) | 30.10±15.20 | 38.15±18.90 | 29.19±25.34 | 28.76±19.47 | 23.74±23.55 |
| NKG2D  (% NK cells) | 71.00±20.00 | 79.02±16.4 | 80.08±15.92 | 70.70±23.40 | 68.32±18.12 |
| NKP30  (% NK cells) | 55.30±13.60 | 52.19±20.44 | 67.25±15.45 | 51.50±21.87 | 56.31±17.80 |
| NKP44  (% NK cells) | 0.30±0.20 | 0.302±0.20 | 0.32±0.283 | 0.203±0.18 | 0.125±0.11 |
| NKP46  (% NK cells) | 43.50±15.40 | 55.22±24.27 | 61.78±12.00 | 46.50±23.19 | 56.95±19.55 |
| KIR3DL1  (% NK cells) | 16.10±5.10 | 16.40±8.80 | 12.80±5.20 | 17.00±6.90 | 13.20±3.54 |
| KIR2DL3  (% NK cells) | 21.50±4.30 | 25.30±9.20 | 21.30±12.70 | 28.60±12.50 | 16.70±5.50 |
| NKG2A  (% NK cells) | 39.40±12.60 | 39.60±17.00 | 38.70±24.90 | 35.80±20.66 | 38.20±14.30 |
| CD158a  (% NK cells) | 20.50±8.40 | 31.50±17.10 | 19.20±10.00 | 17.90±15.30 | 17.80±12.50 |
| CD158b  (% NK cells) | 38.90±10.00 | 43.50±23.90 | 39.70±17.20 | 36.80±16.40 | 48.40±12.30 |
| IFN-gamma  (% NK cells) | 38.60±15.20 | 39.19±20.34 | 40.76±19.47 | 37.74±13.55 | 36.10±15.20 |
| CD107a  (%NK cells) | 5.60±3.10 | 7.58±3.68 | 7.55±3.28 | 5.11±3.19 | 5.57±1.73 |

Data are expressed as means ± SD.
