# Supplementary material for: Natural Killer p46 Controls Hepatitis B Virus Replication and Modulates Liver Inflammation
Source: PLoS One. 2015 Aug 20;10(8):e0135874. doi: 10.1371/journal.pone.0135874 (PMC4546267; doi:10.1371/journal.pone.0135874)
Supplement: S3 Table — (DOC) [file pone.0135874.s003.doc]

Table S3 NK cell expression of NKP46 was abnormal in CHB patients.

| groups | NKP46(%NK cells) |
| --- | --- |
| ALT>100U/L | 42.94±24.28 |
| ALT<100U/L | 57.79±17.04 |
| AST>80U/L | 45.85±23.76 |
| AST<80U/L | 56.82±18.48 |
| HBV DNA<10e5 | 48.38±23.33 |
| HBV DNA>10e5 | 58.74±12.37 |
| HBeAg+ | 50.51±24.02 |
| HBeAg- | 53.71±17.05 |

Data are expressed as means ± SD.
