# Supplementary material for: Natural Killer p46 Controls Hepatitis B Virus Replication and Modulates Liver Inflammation
Source: PLoS One. 2015 Aug 20;10(8):e0135874. doi: 10.1371/journal.pone.0135874 (PMC4546267; doi:10.1371/journal.pone.0135874)
Supplement: S4 Table — (DOC) [file pone.0135874.s004.doc]

Table S4.NK cell expression of CD107a was abnormal in CHB patients.

| groups | CD107a (%NK cells) |
| --- | --- |
| immune-tolerant | 2.92±0.94 |
| immune-activated | 6.8±6.27 |
| HBeAg+ | 2.92±0.94 |
| HBeAb+ | 6.8±6.27 |
| HBV DNA<10e5 | 14.52±19.61 |
| HBV DNA>10e5 | 8.41±10.05 |

Data are expressed as means ± SD.
